# Differentiating rheumatoid and psoriatic arthritis: a systematic analysis of high-resolution magnetic resonance imaging features—preliminary findings

**DOI:** 10.1007/s00256-020-03588-5

**Published:** 2020-08-26

**Authors:** Daniel B. Abrar, Christoph Schleich, Ralph Brinks, Christine Goertz, Matthias Schneider, Sven Nebelung, Philipp Sewerin

**Affiliations:** 1 Medical Faculty, Department of Diagnostic and Interventional Radiology, University Dusseldorf, 40225 Dusseldorf, Germany; 2 Policlinic and Hiller Research Unit of Rheumatology, UKD, Heinrich Heine University Düsseldorf, Moorenstrasse 5, 40225 Dusseldorf, Germany

**Keywords:** Psoriatic arthritis, Rheumatoid arthritis, MRI, PsAMRIS, RAMRIS, Metacarpophalangeal joint

## Abstract

**Background:**

Because of overlapping phenotypical presentations, the diagnostic differentiation of rheumatoid arthritis (RA) and psoriatic arthritis (PsA) remains challenging. Thus, this study aimed to examine the diagnostic value of distinct imaging features obtained by high-resolution 3-T MRI for the diagnostic differentiation.

**Materials and methods:**

Seventeen patients with PsA and 28 patients with RA were imaged at high resolution using 3-T MRI scanners and a dedicated 16-channel hand coil. All images were analyzed according to the outcome measures in rheumatology clinical trials’ (OMERACT) RAMRIS (Rheumatoid Arthritis Magnetic Resonance Imaging Score) and PsAMRIS (Psoriatic Arthritis Magnetic Resonance Imaging Score) for the presence and intensity of synovitis, flexor tenosynovitis, bone edema, bone erosion, periarticular inflammation, bone proliferation, and joint space narrowing. Next, odds ratios (OR) were calculated to determine the strength of the associations between these imaging features, demographic characteristics, and the outcome RA vs. PsA.

**Results:**

PsA could be differentiated from RA by extracapsular inflammatory changes (PsAMRIS sub-score “periarticular inflammation”), with low odds for the presence of RA (OR of 0.06, *p* < 0.01) at all metacarpophalangeal (MCP) joints. A prediction model informed by the items that were strongest associated with the presence of RA or PsA demonstrated excellent differentiating capability with an area under the curve of 98.1%.

**Conclusion:**

High-resolution imaging is beneficial for the identification of relevant imaging features that may assist the clinical differentiation of inflammatory conditions of the hand. At the MCP level, extracapsular inflammatory changes were strongly associated with PsA and may consequently allow the imaging differentiation of PsA and RA.

**Electronic supplementary material:**

The online version of this article (10.1007/s00256-020-03588-5) contains supplementary material, which is available to authorized users.

## Introduction

Rheumatoid arthritis (RA) and psoriatic arthritis (PsA) are chronic inflammatory conditions that cause progressive destruction of cartilage and bone [1]. Even though these entities share pathophysiological features and phenotypical manifestations, several studies hypothesized that RA and PsA differ ultimately in their pattern of joint involvement [2]. This is reflected by the current enthesis organ concept that highlights the role of fibrocartilaginous insertion sites of tendons and ligaments in the pathogenesis of PsA [3, 4]. In contrast, RA is considered a synovial disease with secondary involvement of periarticular insertion sites and ligaments [5].

Despite the fact that imaging plays only a minor role in commonly applied clinical classification schemes [6, 7], characteristic imaging features exist for both entities and may be visualized using radiography, ultrasound, or magnetic resonance imaging (MRI) [8]. MRI can provide crucial information on a range of pathological manifestations such as synovitis, enthesitis, bone edema, erosion, and osteoproliferation [9, 10]. Even though MRI is not used as the primary diagnostic imaging modality for RA or PsA, it is increasingly used to evaluate treatment response. The outcome measures in rheumatology clinical trials (OMERACT) initiative established MRI scores for PsA and RA, i.e. PsAMRIS (Psoriatic Arthritis Magnetic Resonance Imaging Score) and RAMRIS (Rheumatoid Arthritis Magnetic Resonance Imaging Score) [11, 12]. However, despite great progress over the last years in defining typical imaging features, there remains significant overlap between both entities that challenges unambiguous diagnosis [13]. Especially in polyarticular disease, distinction with certainty can be difficult. Consequently, recent studies outlined certain imaging features that might assist differentiation in these “borderline” cases but have not been able to provide measures or means for valid and reliable differentiation [14]. In addition to truly overlapping phenotypical manifestations of the two diseases, this diagnostic limitation may be the result of insensitive imaging modalities. In MRI, field strength needs to be invested adequately to achieve proper image resolution, signal-to-noise ratio, and contrast to best differentiate the disease entities, in particular when imaging delicate structures such as the small finger joints, the entheses, and the nails for subtle changes. This may be hampered when field strength is low (i.e. ≤ 1.5 T) and coils are inadequate [15–17].

In the past, RA and PsA patients have received the same therapy regimes, rendering diagnostic differentiation largely irrelevant. The benefit of a tailored treat-to-target approach for each type of arthritis [18, 19], however, and the development and increasing application of biological disease-modifying drugs (bDMARD) and targeted synthetic disease-modifying drugs (tsDMARDs) has made the diagnostic differentiation between RA and PsA increasingly important to guide treatment in each individual patient.

In the present study, we aimed to systematically assess and quantify the diagnostic value of a range of inflammatory and noninflammatory MRI features obtained at high resolution in differentiating rheumatoid and psoriatic arthritis using clinical classification criteria as reference. We hypothesized that the distribution, severity, and tissue involvement of these imaging features (a) was variable in both entities and (b) could be used to quantitatively assess each imaging feature’s contribution to the diagnosis of RA or PsA.

## Materials and methods

### Study population

Characteristics of the study populations are summarized in Table 1.

**Table 1:** Demographic characteristics of the study populations. For patients with psoriatic (PsA) and rheumatoid arthritis (RA), the mean age in years ± standard deviation (SD) and range, the mean disease duration in years (PsA) and weeks (RA) ± SD and range, and the sex are presented

	PsA patients	RA patients
Population size	17	28
Age (years)	53.7 ± 11.6 (26–72)	55 ± 11.4 (39–74)
Disease duration	2.6 ± 3.3 (1–8 years)	6.3 ± 10.1 (2–23 weeks)
Sex (male/female)	9 males/8 females	9 males/19 females

A total of 17 patients with PsA (mean age 53.7 ± 11.6; range 26–72 years; 9 males, 8 females), who fulfilled the CASPAR criteria [5], had a mean disease duration of 2.6 ± 3.3 years, and suffered from peripheral joint involvement, were prospectively recruited for the “Analysis of the DActylic Melange” (ADAM) research initiative [10]. All patients had failed monotherapy with the conventional synthetic disease-modifying antirheumatic drug (csDMARD) methotrexate (MTX) and were escalated to anti-TNF therapy following an MRI scan.

Additionally, 28 patients with RA (mean age 55.0 ± 11.4 years; range 39–74 years; 9 males, 19 females), who fulfilled the ACR/EULAR 2010 criteria for RA and had a mean disease duration < 6 months (mean 6.3 ± 10.1; range 2–23 weeks), were prospectively recruited from the German ArthroMark initiative cohort [20]. All patients were treated with MTX without any increase in dosage nor change to other medications.

Exclusion criteria for both arms of the total study population were pregnancy, current breastfeeding, age < 18 years, metal implants, implanted medical devices (e.g. ventriculoperitoneal shunts), claustrophobia, asthma, known malignancy, and known osteoarthritis of the hands.

Disease activity was assessed by the Disease Activity Score 28 (DAS28, remission < 2.6, low 2.6 to < 3.2, moderate 3.2 to 5.1, high > 5.1) [21] for RA and the Disease Activity index for PsA (DAPSA, remission 0–4, low 5 to 14, moderate 5 to 28, high > 28) [22] for PsA patients, which are commonly used quantitative measures to assess disease activity and progress in RA and PsA, respectively [23, 24]. In our study, the mean DAS28 was 4.69 ± 0.84 and the mean DAPSA was 26.89 ± 18.23. In addition, serum C-reactive protein (CRP) levels were determined for both cohorts (RA 0.96 ± 0.93 mg/dL; PsA 1.1 ± 1.7 mg/dL).

The present study was approved by the local ethical committee (Ethical Committee of the Medical Faculty of the University of Düsseldorf, Germany, study number: 3828 and 4962R. Trial registration: 2014123117). Written and informed consent was obtained from all patients prior to initiation.

### MRI studies

In PsA patients, MR imaging of the clinically dominant hand was performed using a 3-T MRI scanner (Magnetom Skyra, Siemens Healthineers, Erlangen, Germany). In RA patients, a 3-T MRI scanner (Magnetom Trio, A Tim System; Siemens Healthineers) was used. In both scanners, patients were examined in a prone position with their arm extended overhead and the palm facing down (“superman position”). For high-resolution scanning, a dedicated 16-channel hand coil (3-T Tim receive-only coil, Siemens Healthineers) was used, allowing for a high-resolution imaging over a wider area compared with previous high-resolution strategies.

The imaging protocol was implemented in line with the recommendations of the OMERACT working group [11, 25] and included pre- and post-contrast T1-weighted (T1w) and non-contrast fat-saturated T2w/STIR images in two different orthogonal planes. More specifically, the following sequences of the clinically dominant hand were obtained: coronal short tau inversion recovery (STIR) and T1w turbo spin echo (TSE) sequences. Following intravenous injection of the contrast agent (gadolinium-based, 0.4 mL/kg body weight gadoteric acid [Gd-DOTA], Dotarem, Guerbet Villepinte, France for PsA and [Gd-DTPA], Magnevist; Schering, Berlin, Germany, in RA patients), coronal and transversal T1wTSE sequences with spectral fat suppression were applied. The field of view covered the metacarpophalangeal (MCP) and the proximal and distal interphalangeal (PIP, DIP) joints in PsA patients, and the wrist, the carpus, and the MCP joints in RA patients.

Detailed sequence parameters are given in Table 2.

**Table 2:** Detailed magnetic resonance imaging (MRI) sequence parameters

Sequence		Orientation	TR/TE (ms)	Flip angle (°)	Slice thickness (mm)	FoV (mm × mm)	Acquisition matrix (pixels)	Pixel size (mm/pixel)
T1w TSE	PsA	Coronal	862/27	150	2.5	140 × 140	512 × 512	0.27 × 0.27
	RA		862/27	150	2.5	130 × 130	512 × 512	0.25 × 0.25
T1w TSE + contrast	PsA	Coronal	862/27	150	2.5	140 × 140	512 × 512	0.27 × 0.27
	RA		862/27	150	2.5	130 × 130	512 × 512	0.25 × 0.25
STIR	PsA	Coronal	5560/31	120	2.5	140 × 140	448 × 314	0.31 × 0.41
	RA		5560/31	120	2.5	130 × 130	448 × 314	0.29 × 0.41
T2w TSE fs	PsA	Transversal	5694	89	3.0	160 × 160	512 × 358	0.31 × 0.45
	RA		na	na	na	na	na	na
PD TSE fs	PsA	Sagittal	3150/47	150	2.5	150 × 150	448 × 182	0.33 × 0.82
	RA		na	na	na	na	na	na
T1 TSE fs + contrast	PsA	Transversal	807/16	90	2.5	130 × 130	384 × 288	0.31 × 0.42
	RA		702/16	90	2.5	130 × 130	384 × 288	0.31 × 0.42

Imaging plane, echo and repetition time (TE/TR), flip angle, slice thickness, field of view (FoV), pixel size, and number of slices are given for all sequences (short tau inversion recovery, T2-weighted fat-saturated turbo spin echo (T2w TSE fs), T1w TSE, proton density TSE fs (PD))

### Image analysis

**Fig. 1:**
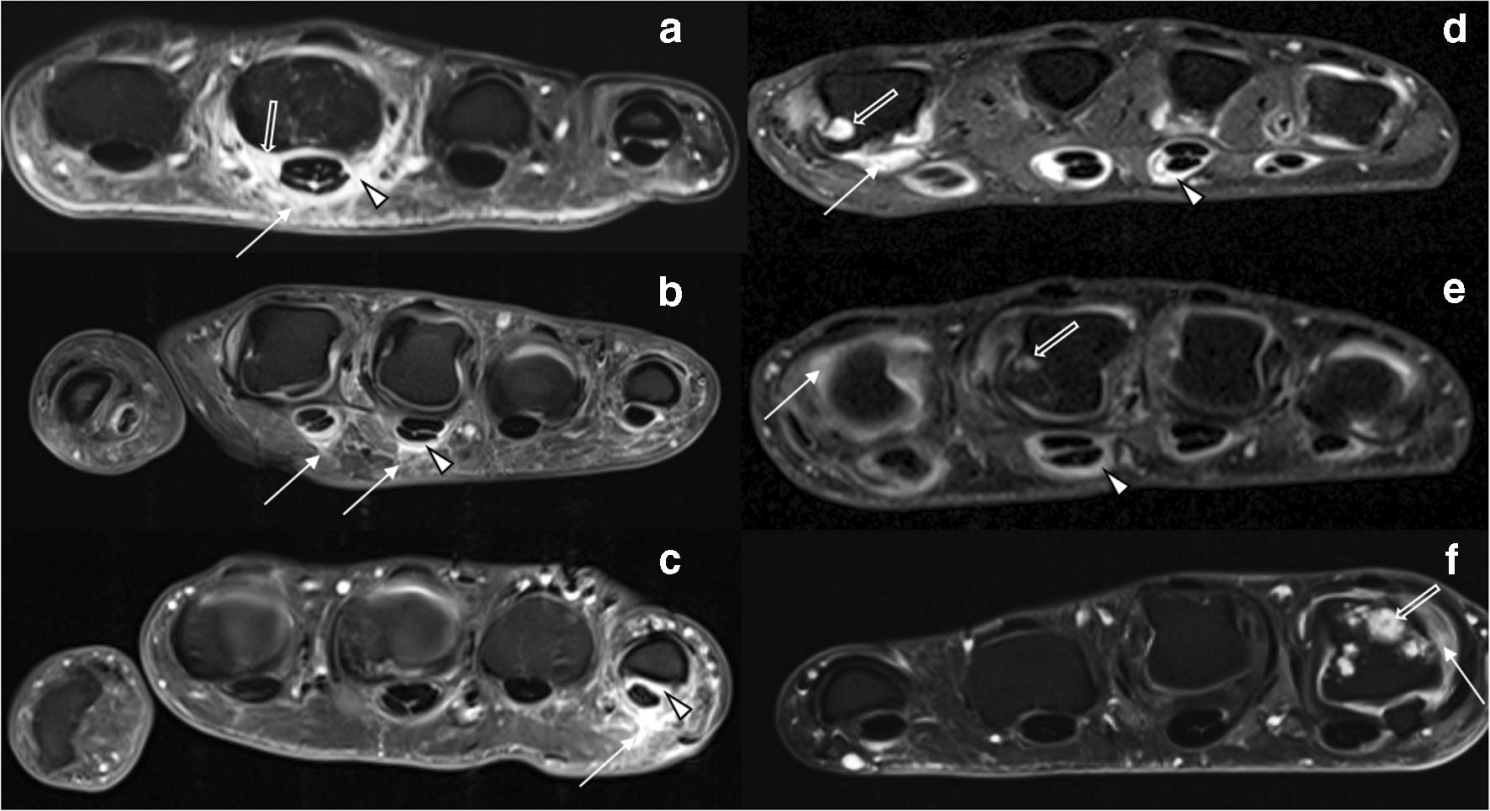
Overview of representative MRI findings in psoriatic arthritis (PsA) and rheumatoid arthritis (RA). Transversal T1w fat-saturated contrast-enhanced sequences of selected MCP joints of three patients each with PsA (**a**–**c**) and RA (**d**–**f**). **a** 39-year-old male. Severe periarticular inflammation (white arrow) with additional flexor tenosynovitis (arrowhead) and synovitis (open arrow). **b** 43-year-old male. Moderate periarticular inflammation (white arrow) with flexor tenosynovitis (open arrow). **c** 39-year-old female. Severe periarticular inflammation (white arrow) with corresponding flexor tenosynovitis (arrowhead). **d** 48-year-old female. Widespread synovitis (white arrow), bone erosions (open arrow), and severe flexor tenosynovitis (arrowhead). **e** 39-year-old male. Bone erosion (open arrow), synovitis (white arrow), and severe flexor tenosynovitis (arrowhead). **f** 43-year-old male. Multiple bone erosions (open arrow) and synovitis (white arrow). Note the absence of periarticular inflammation in **d**–**f** despite significant inflammatory joint changes at the joint level

**Fig. 2:**
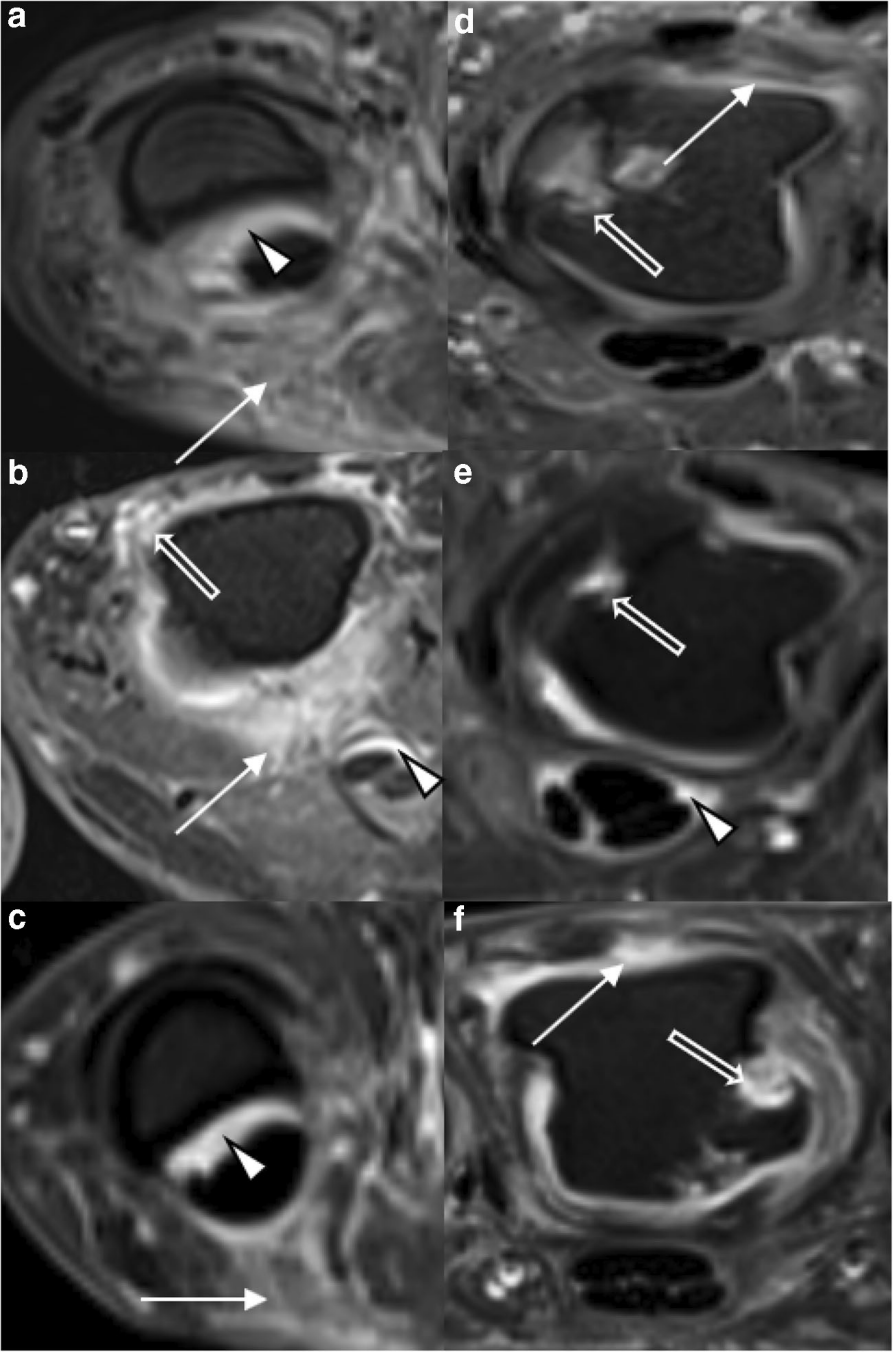
Detailed view of representative MRI findings in psoriatic arthritis (PsA) and rheumatoid arthritis (RA). Transversal T1w fat-saturated contrast-enhanced sequences of selected MCP joints of three patients each with PsA (**a**–**c**) and RA (**d**–**f**). **a** 29-year-old female. Severe periarticular inflammation (white arrow) with additional flexor tenosynovitis (arrowhead). **b** 47-year-old female. Severe periarticular inflammation (white arrow) with synovitis (open arrow) and flexor tenosynovitis (arrowhead). **c** 37-year-old male. Moderate periarticular inflammation (white arrow) with corresponding flexor tenosynovitis (arrowhead). **d** 55-year-old male. Widespread synovitis (white arrow) and multiple bone erosions (arrowhead). **e** 48-year-old female. Bone erosion (open arrow) and slight flexor tenosynovitis (arrowhead). **f** 39-year-old male. Bone erosion (open arrow) and synovitis (white arrow). Note the absence of periarticular inflammation in **d**–**f** despite significant inflammatory joint changes at the joint level

**Fig. 3:**
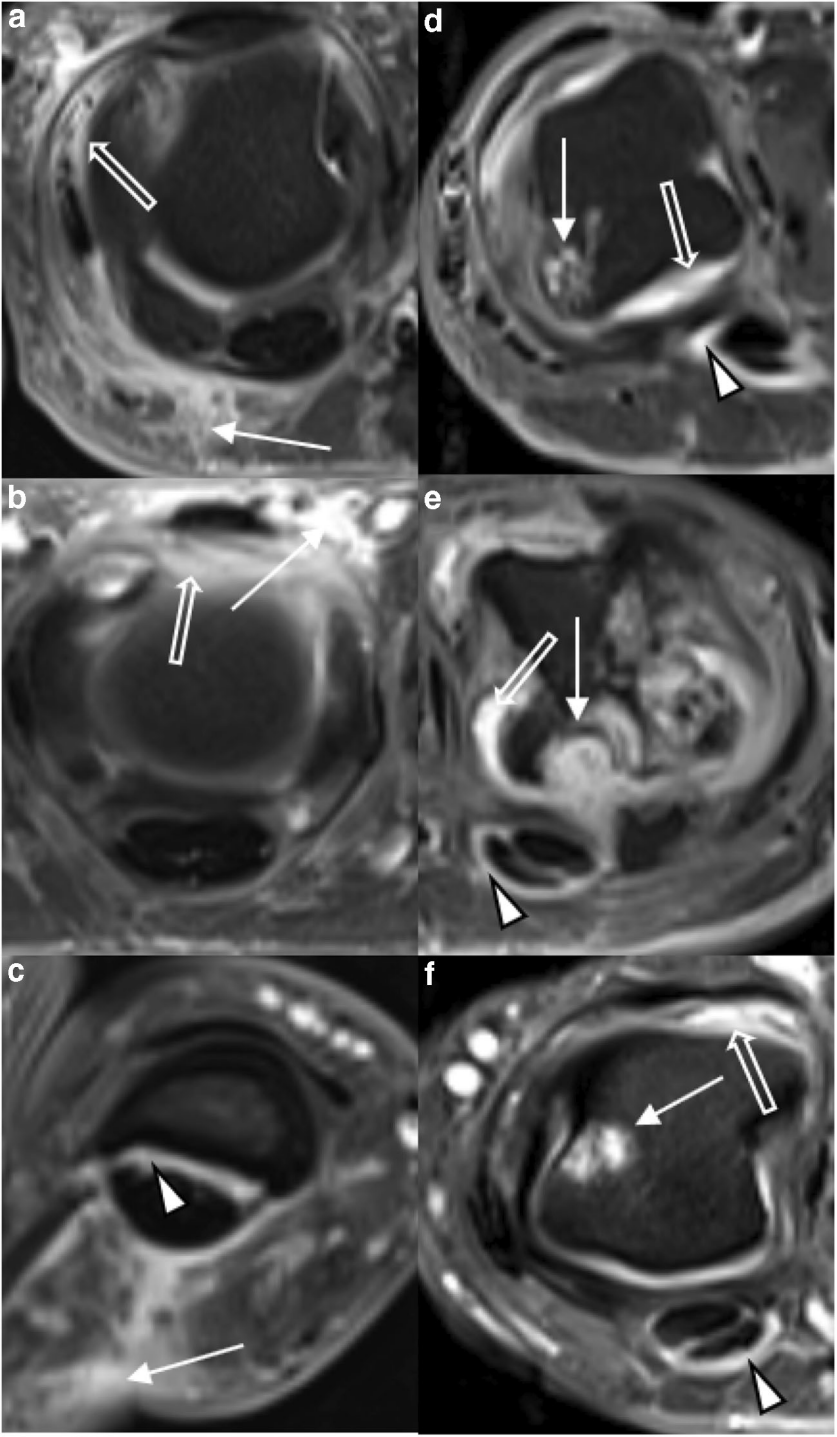
Detailed view of representative MRI findings in PsA and RA. Transversal T1w fat-saturated contrast-enhanced sequences of selected MCP joints of three patients each with PsA (**a**–**c**) and RA (**d**–**f**). **a** 34-year-old male. Severe periarticular inflammation (white arrow) with additional mild synovitis (open arrow). **b** 42-year-old female. Severe dorsal periarticular inflammation (white arrow) with synovitis (open arrow). **c** 44-year-old female. Severe periarticular inflammation (white arrow) with corresponding mild flexor tenosynovitis (arrowhead). **d** 39-year-old male. Widespread synovitis (white arrow) with moderate flexor tenosynovitis (arrowhead) and large bone erosion (white arrow). **e** 41-year-old male. Multiple large bone erosions (white arrow) and severe synovitis (open arrow) with mild flexor tenosynovitis (arrowhead). **f** 56-year-old female. Bone erosion (white arrow) with moderate flexor tenosynovitis (arrowhead) and moderate synovitis (open arrow). Note the absence of periarticular inflammation in **d**–**f** despite significant inflammatory joint changes at the joint level

MR images were randomized and then independently read and analyzed by two radiologists (both trained in musculoskeletal imaging and in PsAMRIS and RAMRIS scoring with 3 (DBA) and 8 (CS) years of experience) according to the OMERACT guidelines [11, 26]. In case of different findings, the raters decided by common agreement with the assisting opinion of a third rater (PS, rheumatologist with 8 years of experience). The raters were blinded to patients’ data and treatment and did not partake in data collection. According to the definitions of the OMERACT guidelines [11, 25], images were evaluated for synovitis (score 0–3), flexor tenosynovitis (score 0–3), periarticular inflammation (score 0 or 1), bone edema (score 0–3), bone erosion (score 0–10), bone proliferation (score 0 or 1), and joint space narrowing (score 0 or 1) of the MCP joints of digits 2–5, with higher scores indicating more severe inflammatory changes. Typical changes are depicted in Figs. 1, 2, and 3.

### Statistical analysis

All statistical analyses were performed by RB using the R project for statistical computing (version 3.5.1, The R Foundation for Statistical Computing, https://www.r-project.org/). For descriptive analysis, the mean, standard deviation, minimum, and maximum were determined. For each item of the scores, age and sex, univariate logistic regression models, odds ratios (OR), and 95% confidence intervals (CI) were calculated. The results of these analyses are shown in a forest plot (Fig. 3). *p* values < 0.05 were considered significant. Based on these data, we set up a prediction model related to age, gender, periarticular inflammation (volar portion of MCP 5), and bone erosion (proximal joint portion of MCP 5) that attempted to correctly identify PsA- and RA-associated imaging features to establish a diagnosis. The resulting coefficients of the prediction model on the link scale of the logistic regression, i.e. log-OR, are given in Supplementary Table 1. Consequently, receiver operating characteristics (ROC) were calculated. Of note, the variables age, gender, periarticular inflammation, and bone erosion were selected due to their OR (strongest and second strongest OR of two different score items) and potential clinical practicability and availability (gender, age, and one particular digit). OR > 1 indicates that the respective variable is associated with the outcome RA. Variables linked to the outcome PsA, or strictly speaking “not RA,” have OR < 1. Confidence bounds for the prediction model were estimated by the bootstrap method [27] (based on *B* = 5000 bootstraps with replacement) and application of the percentile method [28]. Based on the ROC, an area under the curve (AUC) was obtained, which is an index that indicates the diagnostic value of a test (set) and varies from 0.5 (no apparent accuracy) to 1.0 (perfect accuracy) [29]. Inter- and intra-rater reliability were calculated by two-way mixed intra-class correlation coefficients (single-measure intraclass correlation coefficient (sICC) for intra-rater; average-measure ICC (aICC) for inter-rater reliability).

## Results

### Distribution of RAMRIS and PsAMRIS sub-scores inter- and intra-rater reliability

Frequency, distribution, and means as well as standard deviations for each scored item are listed in Table 3. Typical disease-related findings are illustrated in Figs. 1 and 2. sICC and aICC were > 0.9.

**Table 3:** Distribution, frequency, and severity of combined RAMRIS and PsAMRIS sub-scores of metacarpophalangeal (MCP) joint 2–5 in patient cohorts with psoriatic arthritis (PsA) or rheumatoid arthritis (RA). Data are presented as [frequency in %] and median (interquartile range) for the MCP joint of each digit (D2–D5) and overall. *na* not applicable

Item		Joint portion	D2	D3	D4	D5	Overall
Synovitis	PsA	na	[100%] 2 (2–3)	[100%] 3 (2–3)	[100%] 2 (2–3)	[100%] 2 (1–3)	9 (7–11)
	RA	na	[100%] 3 (2–3)	[100%] 3 (2–3)	[100%] 2 (1–3)	[100%] 2 (1–3)	9.5 (7–11)
Flexor tenosynovitis	PsA	na	[100%] 1 (1–1)	[(94%] 1 (1–2)	[94%] 1 (1–1)	[88%] 1 (1–2)	5 (4–6)
	RA	na	[100%] 2 (1–2)	[96%] 1 (1–2)	[86%]) 1 (1–2)	[89%] 1 (1–2)	5 (4–7)
Periarticular inflammation	PsA	Volar	[82%] 1 (1–1)	[76%] 1 (1–1)	[88%] 1 (1–1)	[88%] 1 (1–1)	4 (3–4)
		Dorsal	[65%] 1 (0–1)	[76%] 1 (1–1)	[65%] 1 (0–1)	[82%] 1 (1–1)	4 (2–4)
	RA	Volar	[14%] 0 (0–0)	[7%] 0 (0–0)	[7%] 0 (0–0)	[4%] 0 (0–0)	0 (0–0)
		Dorsal	[46%] 0 (0–1)	[35%] 0 (0–1)	[21%] 0 (0–0)	[21%] 0 (0–0)	1 (0–2)
Bone edema	PsA	Proximal	0	[12%] 0 (0–0)	0	[6%] 0 (0–0)	0 (0–0)
		Distal	[6%] 0 (0–0)	[12%] 0 (0–0)	0	[6%] 0 (0–0)	0 (0–0)
	RA	Proximal	[4%] 0 (0–0)	[11%] 0 (0–0)	0	0	0 (0–0)
		Distal	[4%] 0 (0–0)	[11%] 0 (0–0)	[4%] 0 (0–0)	0	0 (0–0)
Bone erosion	PsA	Proximal	[53%] 0 (0–1)	[47%] 0 (0–1)	[35%] 0 (0–1)	[29%] 0 (0–1)	2 (1–3)
		Distal	[12%] 0 (0–0)	[6%] 0 (0–0)	[6%] 0 (0–0)	[6%] 0 (0–10)	0 (0–0)
	RA	Proximal	[36%] 0 (0–1)	[36%] 0 (0–1)	[18%] 0 (0–0)	[14%] 0 (0–0)	0.5 (0–2.25)
		Distal	[29%] 0 (0–1)	[18%] 0 (0–0)	0	[7%] 0 (0–0)	0 (0–1)
Bone proliferation	PsA	na	[6%] 0 (0–0)	0	[6%] 0 (0–0)	0	0 (0–0)
	RA	na	0	0	0	0	0
Joint space narrowing	PsA	na	[29%] 0 (0–1)	[59%] 1 (0–1)	[65%] 1 (0–1)	[41%] 0 (0–1)	2 (0.25–3.75)
	RA	na	[29%] 0 (0–1)	[50%] 0.5 (01)	[68%] 1 (01)	[0.32%] 0 (0–1)	2 (1–3.25)

### Odds ratios for gender, age, and RAMRIS and PsAMRIS sub-scores for the outcome RA vs. PsA

The odds ratios (OR) for gender, age, and RAMRIS and PsAMRIS items for the outcome RA are presented in Table 4. Periarticular inflammation of the dorsal and volar aspects of MCP 3–5 as well as of the volar aspect of MCP2 were strongly associated with the presence of PsA, as indicated by small ORs (OR < 0.15 [*p* < 0.05]). By trend, periarticular inflammation of the dorsal aspect of MCP2 and bone erosion at the proximal portion of MCP5 were similarly associated with the presence of PsA, yet not statistically significantly. In contrast, bone erosion at the distal portion of MCP3 was associated with the presence of RA, yet not significantly.

**Table 4:** Odds ratios (OR) for gender, age, and individual RAMRIS and PsAMRIS sub-scores with 2.5 and 97.5% confidence intervals (CI) for the outcome RA versus PsA. *JSN* joint space narrowing. Statistically significant values are given in italics and further stratified into *p value < 0.05; **p value < 0.01. *na* not applicable. D2–D5 indicate digits 2–5

Item	D2		D3		D4		D5	
	OR	CI	OR	CI	OR	CI	OR	CI
Gender	2.32	0.73/7.69	2.32	0.73/7.69	2.32	0.73/7.69	2.32	0.73/7.69
Age	1	0.95/1.06	1	0.95/1.06	1	0.95/1.06	1	0.95/1.06
Synovitis	1.36	0.56/3.33	0.7	0.27/1.65	0.72	0.31/1.64	0.94	0.46/1.89
Flexor tenosynovitis	6.08**	1.84/29.19	1.03	0.44/2.49	1.95	0.73/6.16	1	0.44/2.32
Periarticular inflammation								
Volar	0.036**	0.01/0.16	0.02**	0/0.12	0.01**	0/0.06	0.01**	0/0.04
Dorsal	0.47	0.13/1.6	0.17*	0.04/0.62	0.15**	0.04/0.54	0.06**	0.01/0.24
Bone edema								
Proximal	9.85e06	4.79e−206/na	1.51	0.6/7.11	na	na	3.65e−08	na/7.53 e204
Distal	0.5	0.03/1.98	1.14	0.55/2.82	9.85 e06	4.79e−206/na	na	na
Bone erosion								
Proximal	0.68	0.28/1.64	1.16	0.72/2.2	0.52	0.16/1.57	0.4	0.09/1.77
Distal	1.83	0.61/7.75	3.04	0.76/4.8	3.65e−08	na/7.53 e204	1.23	0.11/20.78
Bone proliferation	3.65e−08	na/7.53 e204	na	na	3.65 e−08	na/7.53 e204	na	na
JSN	0.1	0.26/3.81	1.13	0.46/2.95	0.83	0.31/2.19	0.59	0.18/1.85

Throughout, flexor tenosynovitis was strongly and significantly associated with the presence of RA, as indicated by large ORs (OR > 6 [*p* < 0.05]). Other imaging features did not demonstrate strong or significant association with either disease entity.

### Prediction model including four variables

**Fig. 4:**
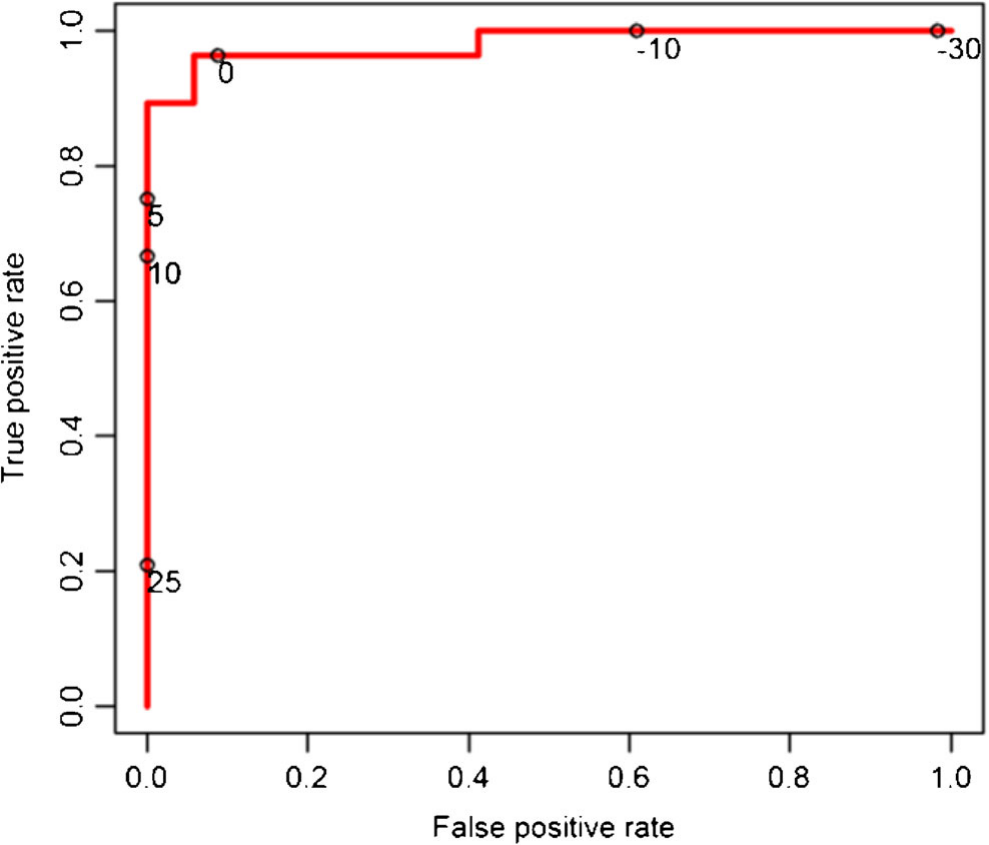
Receiver operating characteristics (ROC) curve to illustrate the diagnostic ability of the calculated prediction model to determine the outcome RA. Given are different discrimination thresholds (circles with adjoined numbers). Area under the curve (AUC) = 98.1%

Gender, age, and the imaging features of erosion of the proximal and periarticular inflammation of the volar joint portion of MCP5 were used to calculate a prediction model for the differentiation between RA and PsA. On the basis of this model, an area under the curve (AUC) of 98.1% (CI = 0.955–1.0) was determined (Fig. 4), therefore accounting for excellent accuracy in distinguishing RA and PsA.

## Discussion

The most important finding of the present study is that the clinical differentiation of inflammatory conditions of the hand may be facilitated by high-resolution MRI features. At the MCP level, extracapsular inflammation was strongly associated with PsA, while flexor tenosynovitis was associated with RA.

Even though the diagnostic differentiation is considered challenging [13], our results indicate that optimized imaging in terms of spatial resolution, signal-to-noise ratio, and contrast can depict subtle changes that may help in clinical decision-making. This is the reason why we used high-resolution MRI studies to explore potentially distinctive imaging features for the differentiation between RA and PsA.

Our results demonstrate that all evaluated imaging features, i.e. synovitis, flexor tenosynovitis, periarticular inflammation, joint space narrowing, bone erosion, bone edema, and bone proliferation can be detected in PsA patients [30]. Except for bone proliferation, all features could also be found in RA patients, which again indicates the considerable morphological overlap between both disease entities. Notably, bone proliferation itself can be considered as a reliable predictor for PsA, even though it could not be included into the presented prediction model due to its rare occurrence in the PsA patient cohort. This may be a consequence of the relatively short disease duration in our patients.

On the other hand, regarding periarticular inflammation, our results show a significant predominance in PsA patients, as reflected by the low odds ratio for the diagnosis RA. Periarticular inflammation is included as a sub-score in the OMERACT PsAMRIS, but not in its RA equivalent, RAMRIS [11, 12, 25]. Both scoring systems are validated semi-quantitative sub-scores for the detection and monitoring of PsA-/RA-related joint changes. By definition, periarticular inflammation is present if increased water content or abnormal contrast-enhancement at extraarticular sites such as soft tissues or entheses can be detected in designated MRI sequences [11, 31]. Previous studies have shown that PsA is an entheseal disease, primarily affecting the so-called “entheseal organs” or the synovio-entheseal complex [4, 32, 33], which consist of tendons and their sheaths as well as ligaments and their insertion sites [34]. Research further indicates that entheseal changes are associated with inflammation of extraarticular tissue [35, 36]; thus, periarticular inflammation is potentially more frequently found in PsA due to its entheseal-driven pathophysiology [37]. Accordingly, in 1995, Jevtic et al. demonstrated that periarticular inflammation is more frequent in PsA than in RA patients, at least at the PIP joint level [38]. Moreover, recent ultrasound and MRI studies have shown that periarticular inflammation is not only a characteristic feature of PsA but is even more frequently detected than in RA [37, 39, 40]. However, literature data are not conclusive as others have reported no difference between RA and PsA concerning extraarticular inflammatory changes and enthesitis [41]. Most likely, these different definitions of morphological changes in both diseases is due to the heterogeneity of PsA and the limited image resolution and signal yield of low-field MRI scanners that may have prevented the detection of subtle changes in small joints. Against this background, our data lend evidence to the use of high-resolution MRI sequences, as in this study, because they seem beneficial for detecting subtle extracapsular changes in efforts to differentiate the two entities.

In the present study, we established a prediction model comprised of demographic characteristics, i.e. age and gender, and imaging features, i.e. periarticular inflammation and erosion at MCP5. The model performed exquisitely well in differentiating both entities as indicated by the AUC value of 98.1, indicating excellent accuracy in differentiating RA from PsA. Even though our model can give an indication of the variables that need particular attention when it comes to the eventual differentiation of both entities, these findings should be treated with caution. First, our sample size was small, and thus the model certainly lacks generalizability until it has been validated in a separate cohort of RA and PsA patients, in particular in terms of different disease stages. Long-standing inflammatory conditions most likely undergo phenotypical changes that are not yet captured by the model. Second, the model was developed on the basis of the best-performing classifiers, which—of course—boosts its diagnostic performance. That is why our findings and the derived prediction model can only be regarded as preliminary and require future studies for their validation. Yet, we consider this methodology a starting point for future research efforts that not only include larger patient cohorts but also variable phenotypical presentations, disease stages, and previous medication regimes. After validation, however, the developed prediction model could be of great clinical significance since the distinction between RA and PsA can be challenging by mere clinical and serological means, especially in borderline cases. With the introduction of treat-to-target therapy regimes in both entities and the increasing development and application of bDMARDs and tsDMARDs, the early diagnostic differentiation between the two diseases has become increasingly important to guide therapy in each individual patient [18, 19].

The only other imaging feature that was significantly associated with one of the two disease entities was flexor tenosynovitis, which was strongly associated with the presence of RA, as already shown in previous studies [10, 40, 42]. Only for MCP2 did we find significant values, while for MCP4, a similar, yet nonsignificant, trend was seen. For MCP2 and 3, however, flexor tenosynovitis was equally distributed between the two entities. One possible explanation for these discrepancies may be the fact that flexor tenosynovitis occurs frequently in both RA and PsA, without a predominant association with one disease entity over the other [31, 43, 44]. Accordingly, this item has been included in both OMERACT MRI scores, RAMRIS and PsAMRIS [11, 25]. Even though Marzo-Ortega et al. detected flexor tenosynovitis more frequently in RA than in PsA, their patient sample was too small to be certain of a significant association [40]. Our own findings demonstrated a similar pattern, with a higher overall prevalence of flexor tenosynovitis in RA patients. Due to conflicting results in different studies including our own, each involving small and selective patient cohorts, no solid conclusions may be drawn as flexor tenosynovitis at the MCP joint level may be associated with both disease entities.

Regarding all other evaluated imaging features, we found an overall homogenous distribution between both entities, with slight differences by trend that are neither statistically nor (most likely) clinically significant.

In addition to mere morphological MRI, compositional imaging techniques have emerged over the last years and have become a powerful tool for diagnosis and monitoring of various musculoskeletal disorders, including inflammatory joint conditions such as RA [45, 46]. Among them, delayed gadolinium enhanced MRI of cartilage (dGEMRIC) is still the gold-standard technique, even though techniques that do not rely on the application of intravenous contrast agents such as T2/T2* mapping or sodium imaging are of increasing scientific and clinical interest [47–50]. However, research on compositional MRI in PsA is sparse, with yet only one single study published [51]. In future studies, nevertheless, compositional MRI techniques could potentially further facilitate the characterization and distinction of RA and PsA.

When interpreting our results, the following limitations must be considered.

First and foremost, both patient cohorts were small and, due to our study’s exploratory design, our results can only be considered as preliminary and need further validation in future studies with larger study populations. Secondly, our PsA cohort may not be representative of the full spectrum of disease stages because all included PsA patients suffered from severe dactylitis in at least one finger. As dactylitis is associated with high disease activity [36], the imaging correlates identified in this study may be predisposed towards higher disease severity, thereby rendering the incidence and severity of enthesitis and periarticular inflammation overly high. Similarly, flexor tenosynovitis may also be overrepresented since it is known to be associated with dactylitis, too [36, 52]. Third, the mean disease duration of both study cohorts differed substantially. With a mean disease duration of 6 and 160 weeks, we compared (very) “early” RA to “long-standing” PsA. Of note, current guidelines define “early” RA in terms of a disease duration of less than 12 months, while “early” PsA is defined as a disease duration of less than 24 months [53, 54]. Thus, the comparability of both cohorts and their respective imaging features in terms of frequency, distribution, and severity is certainly limited. However, periarticular inflammation is a sign of acute inflammation and therefore not limited to late stages of the disease. Since periarticular inflammation is the most predictive feature of our investigation and both patient cohorts had overall similar disease activity as quantified by DAS28 and DAPSA, this limitation seems relevantly attenuated; nonetheless, our findings are only preliminary until confirmed in patient cohorts with equal disease durations. Fourth, we only assessed the imaging features at the MCP joint level. Most likely, the diagnostic differentiation of not just RA vs. PsA but also each entity’s phenotypical manifestation needs to focus on joint levels beyond such as the interphalangeal and intercarpal joints. Future research ought to be directed at quantitatively defining the imaging features there, too. Further, despite the fact readers had been blinded to patient data, different fields-of-view of certain MRI sequences might have corrupted proper blinding. The potential bias was mitigated by the fact that (i) the MCP joints selected for quantitative imaging feature assessment had been imaged using similar sequence and acquisition parameter settings, thereby allowing comparative evaluation, and that (ii) two independent readers analyzed all images in a coherent and standardized manner using the scoring systems of the official OMERACT guidelines. The fact that both readers’ performance was characterized by high inter-reader reliability pays testimony to the validity of their comparative evaluation at the MCP joint level. Additionally, our results have a limited generalizability because of the different prior therapeutic regimes, yet the clinical presentation of increased disease activity was overall comparable. Therefore, we consider the results to be representative of a clinical setting, even though—admittedly—the methodological inaccuracy makes direct comparisons difficult and different types of DMARDs and different treatment durations potentially change intensity, distribution, and quality/dimension of disease-related imaging sub-scores. Last, it is of relevance that MRI can support, but not replace, clinical assessment in the differentiation of inflammatory arthritis. Once the developed prediction model has been validated and substantiated in larger studies and proven to perform well in clinical practice, the clinical differentiation of inflammatory joint diseases may be greatly aided by MRI.

## Conclusion

High-resolution imaging based on optimized sequence protocols, adequate magnetic field strengths, and dedicated coil technology is beneficial for the identification of relevant imaging features that may support the clinical differentiation of inflammatory conditions of the hand. At the MCP level, periarticular inflammation was strongly associated with PsA and may consequently guide diagnostic decision-making when it comes to differentiating PsA and RA.

## Electronic supplementary material

ESM 1 (DOCX 15.6 kb)

## Data Availability

The datasets used and/or analyzed during the current study are available from the corresponding author upon reasonable request.
