# Supplementary material for: Differentiating rheumatoid and psoriatic arthritis: a systematic analysis of high-resolution magnetic resonance imaging features—preliminary findings
Source: Skeletal Radiol. 2020 Aug 26;50(3):531–41. doi: 10.1007/s00256-020-03588-5 (PMC7811987; doi:10.1007/s00256-020-03588-5)
Supplement: Supplementary file 1 — (DOCX 15.6 kb) [file 256_2020_3588_MOESM1_ESM.docx]

| Coefficients | Estimate | Standard error | z-value | Pr (>\|z\|) |
| --- | --- | --- | --- | --- |
| (Intercept) | -9.34 | 8.04 | -1.16 | 0.25 |
| Age [years] | 0.21 | 0.16 | 1.29 | 0.20 |
| Sex [male/female] | 22.07 | 4680.84 | 0.01 | 1.00 |
| Periarticular Inflammation D5 volar | -26.83 | 4680.84 | -0.01 | 1.00 |
| Bone Erosion D5 proximal | -1.26 | 2.01 | -0.62 | 0.53 |

**Supplementary Table** **1**: Given are the coefficients (age, sex, periarticular inflammation of the volar, and bone erosion of the proximal portion of the MCP joint of the fifth digit) based on the results of the univariate logistic regression. Positive estimates indicate the diagnosis RA and negative estimates indicate the diagnosis PsA. Dispersion parameter for binomial family was taken to be 1.
